# Dose reduction does not impact precision of CT-RSA in tibial components in total knee arthroplasty: a randomized controlled trial

**DOI:** 10.1186/s13018-026-06801-8

**Published:** 2026-03-16

**Authors:** Fredrik Bru, Lars H. W. Engseth, Are Hugo Pripp, Anselm Schulz, Tommy Frøseth Aae, Vigdis Schnell Husby, Otto Schnell Husby, Stephan M. Röhrl, Frank-David Øhrn

**Affiliations:** 1 Orthopaedic Department, Nordmøre and Romsdal Hospital (SNR), Møre and Romsdal Hospital Trust, Hjelset, Norway; 2 Division of Orthopaedic Surgery, Oslo University Hospital Ullevål (CIRRO), Oslo, Norway; 3 Faculty of Medicine, University of Oslo, Oslo, Norway; 4 Oslo Centre of Biostatistics and Epidemiology, Research Support Services, Oslo University Hospital, Oslo, Norway; 5 Faculty of Health Sciences, OsloMet – Oslo Metropolitan University, Oslo, Norway; 6 Department of Radiology and Nuclear Medicine, Oslo University Hospital Ullevål, Oslo, Norway; 7 Department of Neuromedicine and Movement Science (INB), Faculty of Medicine and Health Sciences, NTNU Norwegian University of Science and Technology, Trondheim, Norway; 8 The Clinical Research Unit, Møre and Romsdal Hospital Trust, Ålesund, Norway; 9 Faculty of Medicine and Health Sciences, Department of Health Sciences, NTNU Norwegian University of Science and Technology, Ålesund, Norway

## Abstract

**Purpose:**

Radiostereometric analysis (RSA) has been the gold standard for implant migration analysis for decades. Our group previously demonstrated in a porcine cadaver model that CT-RSA precision was non-inferior by dose reduction. The aim of this study was to investigate whether this finding applied in a clinical setting.

**Methods:**

This study is part of the Cless*TKA* trial comparing a novel 3D printed uncemented medially stabilized total knee arthroplasty (TKA) with Tritanium TKA. Fifty patients were recruited (GMK Sphere *n* = 26, Tritanium *n* = 24). All surgeries were performed with mechanical alignment between January and June 2023. CT acquisitions were performed postoperatively within 2 days (standard dose 0.05 mSv, *n* = 49) and at 1 year (low dose 0.01 mSv, *n* = 47) using a GE Revolution scanner. CT-RSA analyses of tibial components were performed with Sectra CTMA software by a certified assessor. The primary endpoint was difference in precision (mean) of maximum total point motion (MTPM) between dose levels, with an equivalence interval of 0–0.1 mm.

**Results:**

Mean difference (95% CI) in MTPM under zero motion circumstances between standard and low dose was 0.007 mm (− 0.018 to 0.032). No clinically meaningful differences were found for migration or rotations. Centre-of-mass analysis showed one significant but clinically irrelevant posterior translation (0.013 mm).

**Conclusion:**

The findings confirm that reducing the effective radiation dose from 0.05 mSv to 0.01 mSv does not compromise the precision of CT-RSA for tibial components in TKA. The results further support the use of low-dose CT-RSA protocols in clinical studies, enabling substantial reduction in patient radiation exposure without loss of precision.

**Trial registration:**

Clinicaltrials.gov identifier NCT05651009. Initial release of the study was November 23, 2022. At that time, the software CTMA (Sectra) did not provide the very important Maximum Total Point Motion (MTPM), this was introduced in a later update of the CTMA. Hence there was change in the original protocol retrospectively.

## Introduction

Total knee arthroplasty (TKA) is a well-established treatment for end-stage knee osteoarthritis. A subset of patients experiences persistent postoperative pain and reduced function [1, 2]. Early detection of excessive implant migration is vital, as it is a strong predictor of long-term loosening and revision surgery [3]. For more than four decades, radiostereometric analysis (RSA) has been the gold standard for measuring implant migration [4–6]. Computed tomography-based RSA (CT-RSA) has emerged as a promising alternative to conventional RSA, offering comparable precision in migration measurements for hip, shoulder and knee implants [7–10]. Unlike RSA, CT-RSA does not require tantalum markers, specialized imaging setups, computer aided design or reversed engineered models. Furthermore, it can be performed using standard CT scanners available in most hospitals. A key limitation, however, has been concerns about the higher radiation dose associated with repeated CT imaging compared to RSA, given the established dose-response relationship between ionizing radiation and cancer risk [11].

In a recent porcine cadaver study on tibial implants, we demonstrated that CT-RSA performed with a 75% reduction in radiation dose maintained precision comparable to that of a standard-dose protocol [12]. The present study aims to validate these findings in vivo by evaluating the precision of low-dose CT-RSA in patients undergoing primary TKA. We hypothesized that low-dose CT-RSA maintained clinically equivalent precision to standard-dose CT-RSA, thereby supporting its use in clinical migration studies while minimizing patient radiation exposure.

## Methods and materials

### Study design and setting

This study was conducted as part of the double-blinded randomized controlled trial “Comparison of the in vivo stability of 2 cementless TKA designs using CT micromotion analysis - A randomized controlled trial” (Cless*TKA*), investigating implant migration in TKA [13]. The trial compared a novel 3D-printed uncemented medially stabilized TKA (GMK Sphere 3D metal, Medacta International, Switzerland) with a well-documented 3D-printed uncemented cruciate-retaining TKA (Triathlon Tritanium CR, Stryker, Mahwah, USA) [14, 15]. The sample size calculation was thus based on the migration analyses and the expected difference in migration between the implants, rather than on the precision of the measurement method used. The surgeries were performed at Kristiansund Hospital, Møre and Romsdal Hospital Trust, Norway, between January and June 2023. All CT acquisitions were performed at the same location between January 2023 and June 2024. A user representative that was not part of the study was appointed and had the opportunity to comment on the protocol.

### Participants

50 patients scheduled for primary TKA due to end-stage osteoarthritis (Kellgren & Lawrence 3–4) (Table 1) were enrolled in the Cless*TKA*, unless they met the exclusion criteria of the study (Table 2) [13]. Patients were randomized into a study group (GMK Sphere, *n* = 26) and a control group (Triathlon Tritanium CR, *n* = 24) using the EFORSK software (Unit for Applied Clinical Research, NTNU, Norway) with stratification by sex and varying block sizes. Randomization was performed no more than 1 week before surgery by the project leader (FDØ) and concealed until allocation. FDØ had no access to, and could thus not affect the randomization algorithm in any way. All procedures were performed with concomitant patella resurfacing, using mechanical alignment and without tourniquet, by two experienced orthopaedic surgeons (FDØ/OSH) consistently working together. All complications were accounted for.

**Table 1:** Demographics of the patients included in the study

Variables (*n* = 50)	Tritanium (*n* = 24)	GMK Sphere (*n* = 26)
Age (years, mean, 95% CI)	64.2 (61.1–67.3)	66.8 (64.5–69.2)
HKA (°, 95% CI)	175.4 (172.7–178.1.7.1)	176.1 (174.0–178.2.0.2)
BMI (kg/m^2^, 95% CI)	29.4 (27.8–31.0)	28.3 (26.8–29.9)
Left/right (n)	12/12	11/15
Male/female (n)	10/14	11/15
Implant (n)	24	26

CI=Confidence Interval, HKA = Hip-Knee-Ankle angle, BMI=Body Mass Index

**Table 2:** Exclusion criteria of the ClessTKA study

Exclusion criteria
Preoperative severe deformity (HKA ≥ 5° valgus or > 15° varus) on full-length weight-bearing radiograph
Preoperative flexion contracture > 15°
Age < 50 or > 75 years at time of surgery Use of walking aids due to other musculoskeletal or neuromuscular problems
Preoperative diagnosis other than OA or avascular necrosis
Revision arthroplasty
BMI > 35
Lateral collateral ligament-deficient knee
Previous knee joint infection

HKA = Hip-Knee-Ankle angle, OA=Osteoarthritis, BMI=Body Mass Index

## CT acquisitions

Double CT acquisitions were performed at two postoperative time points using two different effective doses [16]:

1. *Baseline*—within 2 days of surgery (standard dose: tube current 160 mA, rotation time 0.5 s, scan length 180 mm, tube voltage 120 kV, effective dose 0.05 mSv, *n* = 49).
2. *Follow-up*—at 1 year postoperatively (low dose: tube current 40 mA, rotation time 0.5 s, scan length 180 mm, tube voltage 120 kV, effective dose 0.01 mSv, *n* = 47).

**Fig. 1:**
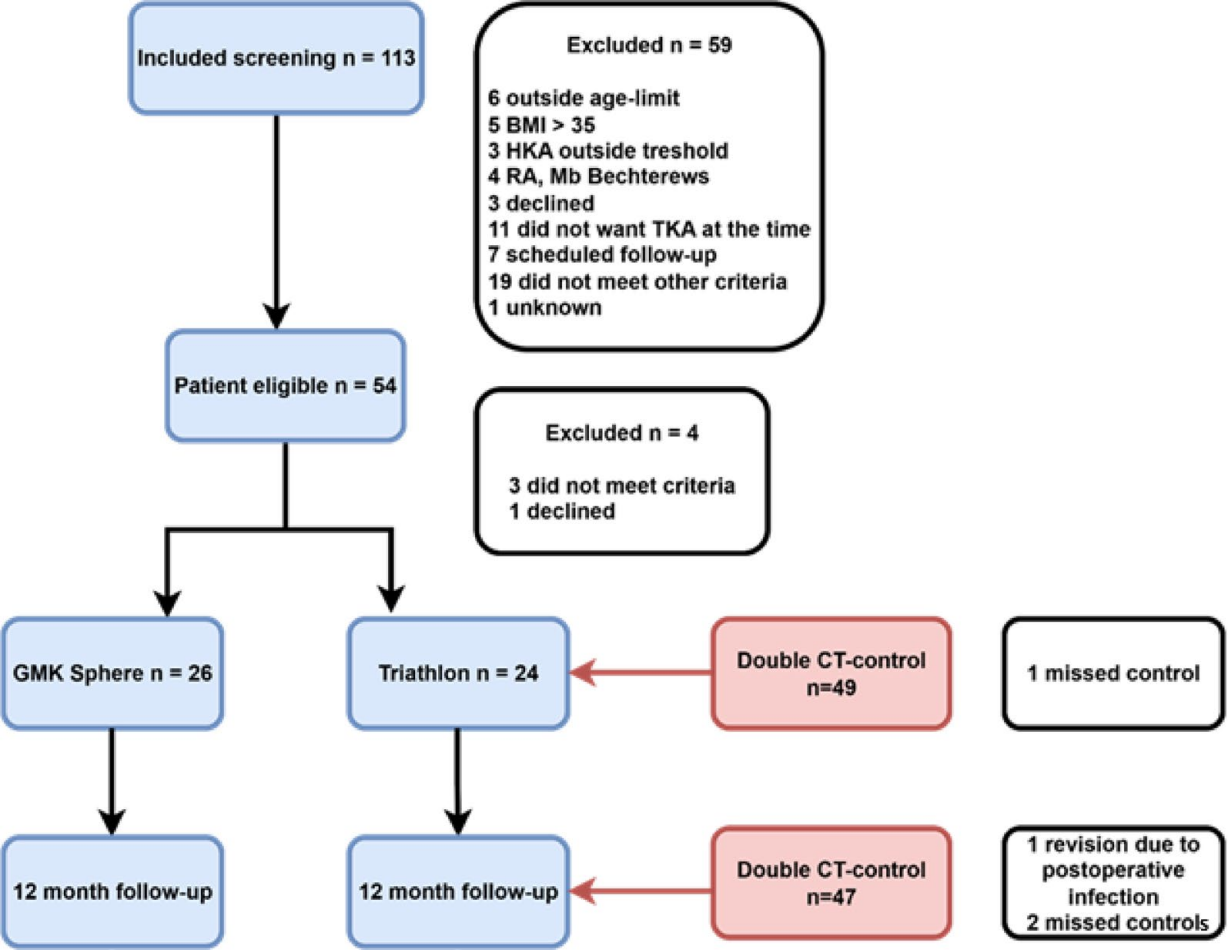
Flow chart of the study. BMI=Body Mass Index, HKA = Hip-Knee-Ankle angle, RA= Rheumatoid Arthritis

All scans were obtained using a GE Revolution CT scanner (GE Healthcare, Chicago, USA). Reconstruction was performed with a Bone Plus metal artefact reduction (MAR) algorithm. Between each double scan, patients moved their knee and the scanner was reset (Fig. 1).

### CT-RSA analysis

**Fig. 2:**
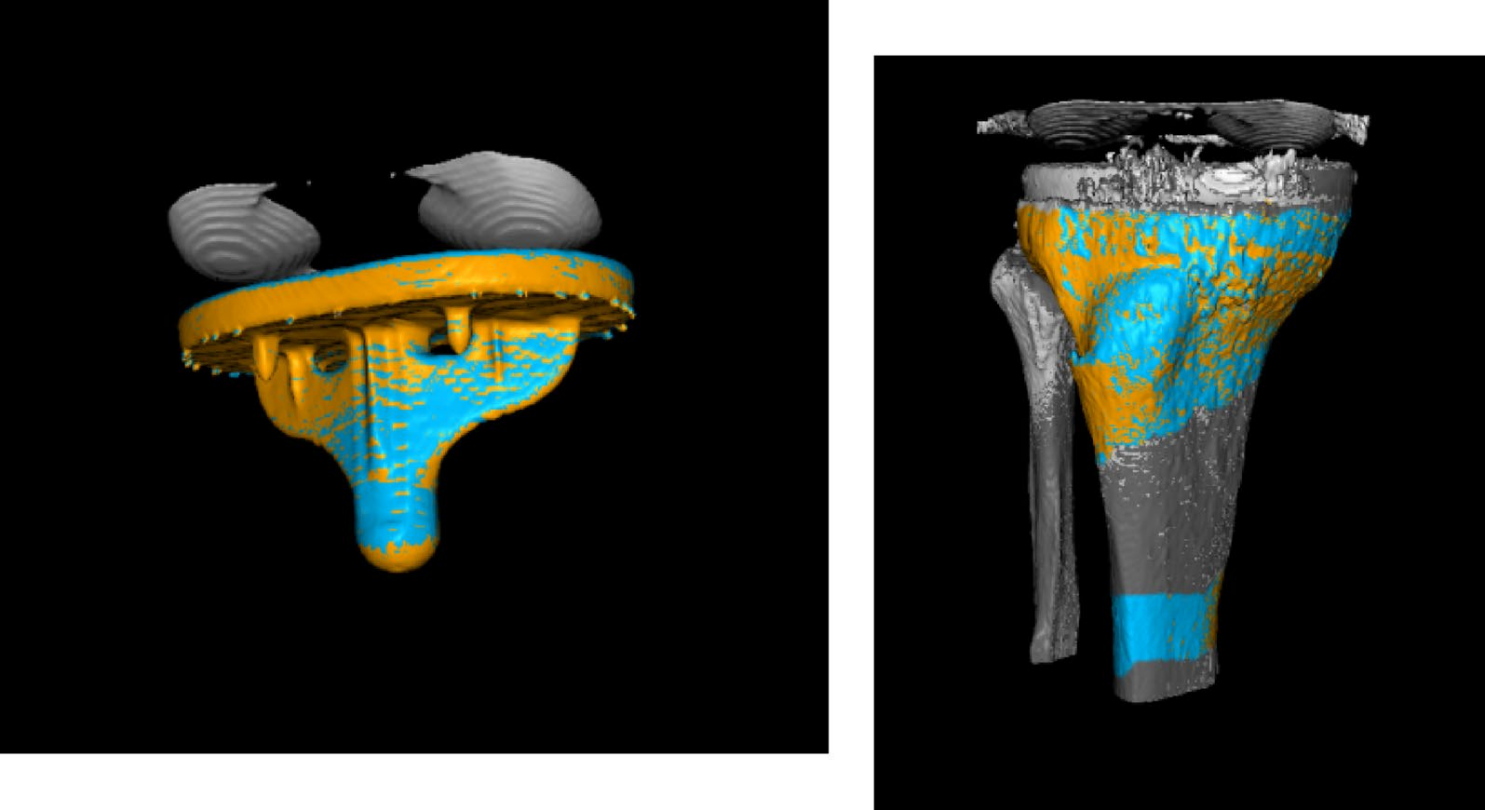
Double click marking of the tibial implant and selective bone marking of the tibia

**Fig. 3:**
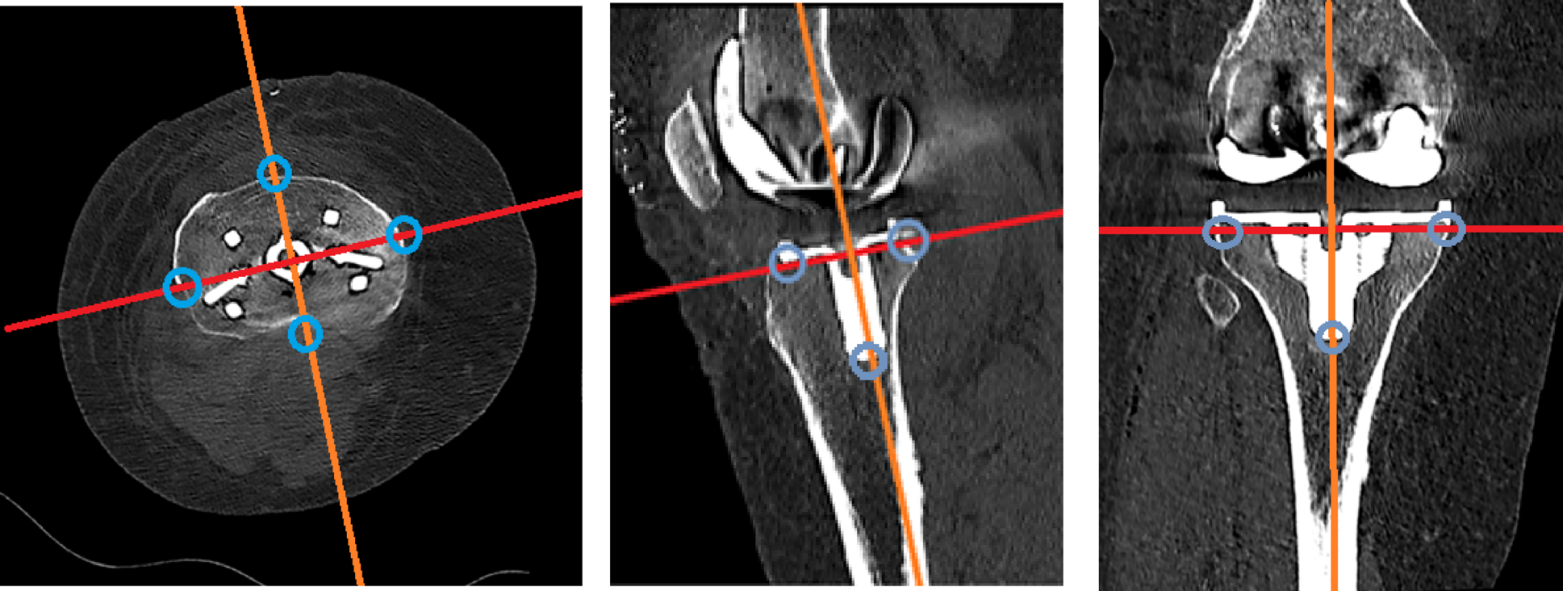
Peripheral points: tip, anterior, posterior, medial and lateral. Multiplanar reconstruction axis set before each analysis

CT-RSA migration measurements were performed using the CT Micromotion Analysis (CTMA) software (Sectra AB, Linköping, Sweden) with Hounsfield Unit (HU) values of 150 for bone and 3000 for metal. The bone was marked selectively, whereas the tibial implants were marked using the double click function of the software (Fig. 2). The standard Digital Imaging and Communication in Medicine (DICOM) coordinate system was used. All multiplanar axes were the same and were set before the analysis. Peripheral points named tip, anterior, posterior, medial and lateral were created (Fig. 3). For each tibial component, the following were recorded:

*Primary endpoint* Maximum total point motion (MTPM), a vector measured in millimetres representing the point of the implant moving the most between the time points [4].

*Secondary endpoints* centre-of-mass (COM) and peripheral points translations and rotations in medial-lateral, proximal-distal and anterior-posterior directions.

Analyses were performed by a CTMA-certified author (FDØ) who was not blinded to implant nor dosage level. All other authors, the patient, study nurse and biostatistician remained blinded during data collection and analysis.

### Statistical analysis

Analyses were performed using Stata version 17 (StataCorp LLC, College Station, TX, USA). Means and 95% confidence intervals (95% CI) were calculated for the variables for standard- and low-dose protocols, and named the precision. The primary outcome measure was the *difference* in MTPM precision between standard- and low-dose CT-RSA. An equivalence margin of 0–0.10 mm was predefined [4, 12, 17], with differences exceeding this considered clinically relevant. Linear mixed models were used, with dose level as a fixed factor and patient ID as a random factor. Standard deviations (SD) of the variables of standard- and low-dose protocols were also calculated. A two-sample F test for equality of variances using the Stata code sdtest, was conducted. Statistical significance was defined as *p* < 0.05.

## Results

**Fig. 4:**
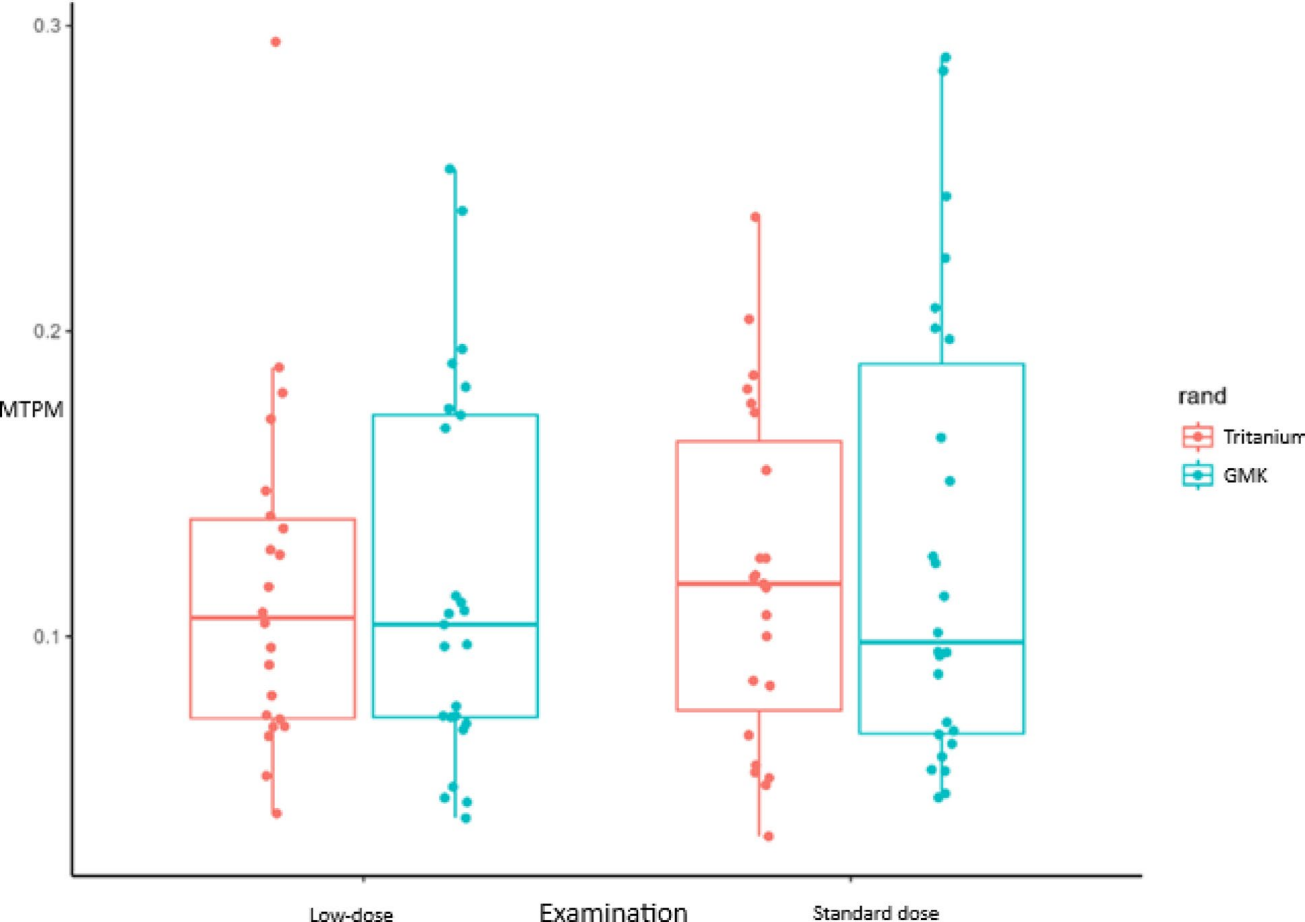
MTPM shown in boxplot format. Examination (low-dose and standard-dose) on the x-axis and MTPM (mm) on the y-axis

**Fig. 5:**
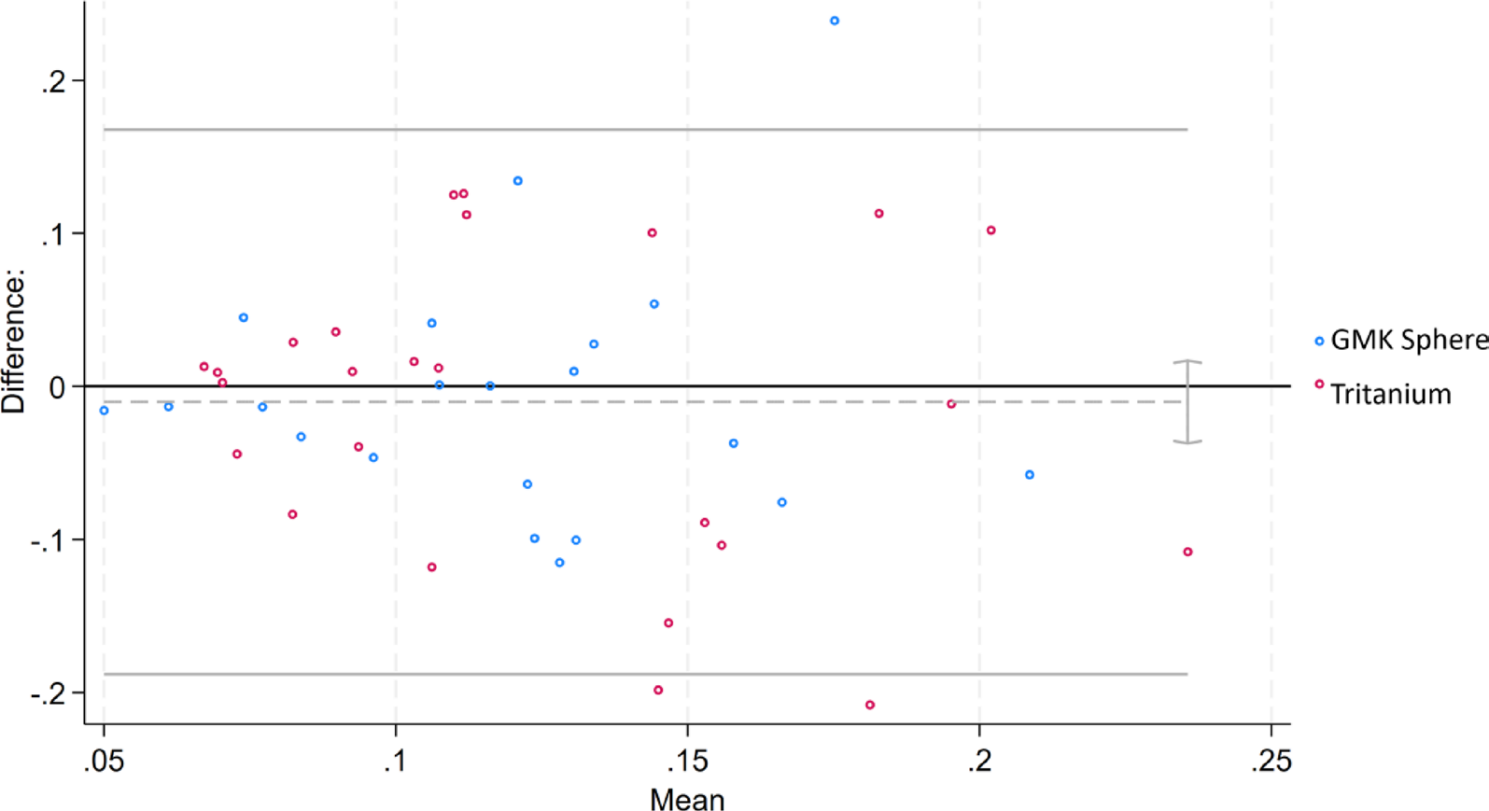
Difference if MTPM shown in Bland-Altman plot. Mean on the x-axis and difference on the y-axis

49 patients completed the baseline (standard dose) and 47 patients the 1-year (low dose) CT acquisitions and were thus included in the precision analysis (Fig. 1). The comparison between standard-dose and low-dose CT-RSA showed a mean difference in MTPM precision of 0.007 mm (95% CI −0.018 to 0.032, *p* = 0.576), well within the predefined equivalence margin of 0–0.10 mm (Figs. 4 and 5). The mean MTPM was 0.117 (0.10 to 0.12) mm for the standard dose, and 0.124 (0.11 to 0.14) mm for the low dose; the SD of both was 0.12 mm. The SD test gave a p value of 0.452. No statistically significant differences in MTPM precision were found between the two implant groups (GMK Sphere vs. Tritanium), with a mean difference of 0.006 mm (95% CI −0.019 to 0.030). Mean and mean difference of segmental translations and rotations, and peripheral points are shown in Table 3.

**Table 3:** Mean and mean difference of segmental translations and rotations, and peripheral points

Variable	Mean standard dose (mm)	Standard deviation	Mean low dose (mm)	Standard deviation	Mean difference (mm)	95% CI
Segmental translation, mm						
Medial	0.00 (−0.01 to 0.01)	0.02	0.00 (−0.01 to 0.01	0.03	−0.001	0.00 to 0.01
Proximal	0.00 (−0.01 to 0.02)	0.05	0.01 (−0.01 to 0.02	0.05	0.002	−0.02 to 0.02
Posterior	−0.01 (−0.02 to −0.00)	0.02	0.00 (−0.01 to 0.01)	0.03	0.013	0.00 to 0.02
Segmental rotation, mm						
Transversal	0.04 (0.00 to 0.01)	0.13	0.01 (−0.02 to 0.05)	0.13	−0.027	−0.08 to 0.02
Internal	−0.01 (−0.03 to 0.01)	0.06	0.00 (−0.02 to 0.02)	0.07	0.012	−0.01 to 0.04
Varus	−0.00 (−0.02 to 0.02)	0.06	0.00 (−0.01 to 0.02)	0.07	0.005	−0.02 to 0.03
Translations of the peripheral points, mm						
Tip						
Medial	0.00 (−0.01 to 0.01)	0.04	−0.00 (−0.01 to 0.01)	0.04	0.04	−0.02 to 0.01
Proximal	−0.00 (−0.02 to 0.01)	0.05	0.00 (−0.01 to 0.02)	0.05	0.005	−0.01 to 0.02
Posterior	0.01 (0.00 to 0.03)	0.06	0.01 (−0.01 to 0.02)	0.05	−0.004	−0.03 to 0.02
Medial						
Medial	0.00 (−0.01 to 0.01)	0.02	0.00 (−0.01 to 0.05)	0.03	0.000	−0.01 to 0.01
Proximal	0.00 (−0.02 to 0.01)	0.05	0.01 (−0.01 to 0.02)	0.06	0.006	−0.01 to 0.03
Posterior	−0.01 (−0.03 to 0.00)	0.04	0.00 (−0.02 to 0.01)	0.06	0.011	−0.01 to 0.03
Lateral						
Medial	0.00 (−0.01 to 0.01)	0.02	0.00 (−0.01 to 0.01)	0.03	0.001	−0.01 to 0.01
Proximal	0.00 (−0.02 to 0.02)	0.05	0.00 (−0.02 to 0.02)	0.07	0.000	−0.03 to 0.03
Posterior	−0.01 (−0.03 to 0.00)	0.07	0.01 (−0.01 to 0.02)	0.05	0.020	0.00 to 0.04
Anterior						
Medial	0.00 (−0.01 to 0.00)	0.03	0.00 (−0.01 to 0.01)	0.03	0.002	−0.01 to 0.01
Proximal	−0.01 (−0.03 to 0.01)	0.07	0.00 (−0.02 to 0.02)	0.07	0.013	−0.01 to 0.04
Posterior	−0.01 (−0.02 to −0.00)	0.03	0.00 (−0.01 to 0.01)	0.04	0.014	0.00 to 0.03
Posterior						
Medial	0.00 (0.00 to 0.01)	0.03	0.00 (−0.01 to 0.01)	0.05	−0.004	−0.02 to 0.01
Proximal	0.02 (0.00 to 0.04)	0.07	0.01 (−0.01 to 0.02)	0.07	−0.013	−0.04 to 0.01
Posterior	−0.01 (−0.02 to 0.00)	0.03	0.00 (−0.01 to 0.01)	0.04	0.014	−0.00 to 0.03

### Complications

One patient in the study group underwent revision due to a deep postoperative infection and was therefore excluded from further analysis at 1 year. In the control group, 1 patient required debridement and irrigation for a postoperative haematoma. Postoperative stiffness requiring manipulation under anaesthesia occurred in 1 patient in the study group and in 2 patients in the control group. Minor wound problems and removal of palpable retained sutures from the knee capsule were noted in a few patients in both groups; all resolved without further intervention.

### Missing data

Double examinations with the standard dose were performed postoperatively, and those with the low dose at 1 year. The patient who developed infection and was excluded from the study lacked low-dose data. In addition, one patient had missing double examinations postoperatively, and two patients at the 1-year follow-up. Details are presented in Fig. 1.

## Discussion

The main finding of this clinical study is that a 75% reduction in effective radiation dose, from 0.05 to 0.01 mSv, does not affect the precision of CT-RSA for tibial components in TKA. These results confirm our previous findings from a porcine cadaver model in a clinical setting, and indicate that low-dose CT-RSA can be implemented without compromising measurement reliability [12, 17].

Our equivalence margin of 0–0.10 mm was based on established thresholds in RSA literature, where increased migration of more than 0.2 mm between 1 and 2 years predicts a higher risk of aseptic loosening [4, 12, 17]. The observed mean difference in MTPM precision (0.007 mm) was far below this margin and statistically non-significant. This supports the clinical feasibility of low-dose CT-RSA for migration monitoring in TKA [12].

The precision values observed in this study are comparable to, or better than, those reported for conventional RSA in tibial components [7]. CT-RSA offers several practical advantages: it does not require tantalum markers, specialized calibration cages, or CAD models, while CT scanners are widely available in hospitals. Furthermore, analysis is less dependent on radiographic positioning and marker visibility, both of which are potential limitations in RSA. The precision described in this study matches the level reported in the only other published study of clinical CT-RSA performed on TKA [18], in which V3MA, not CTMA software, was used [18, 19].

Radiation exposure has been a main argument against the adoption of CT-RSA in clinical research. However, the effective dose in our low-dose protocol (0.01 mSv) is similar to that of a standard RSA examination (0.10–0.15 mSv), especially when accounting for the repeated exposures sometimes required in RSA due to inadequate marker visibility [7]. Given the well-documented dose-response relationship between ionizing radiation and cancer risk, even modest dose reductions are desirable [11].

The only statistically significant difference we observed was a small increase in posterior COM translation at low dose (0.013 mm), which is well within the range of measurement error and not clinically relevant. This finding may reflect normal variability between repeated acquisitions rather than a systematic dose-related effect.

In a recent publication from the Cless*TKA* study using the same patient population, we investigated the precision of CT-RSA on femoral implants [13]. Femoral and tibial components have different geometrical shapes that may affect the precision differently. Because of this, CT-RSA of femoral components have acceptable, though lower precision than the findings in the present study [13]. In TKA, the tibial component is more prone to loosening than the femoral component. Establishing the precision of CT-RSA in tibial components at different dosage levels in a clinical study therefore represent a huge and important step towards proving that CT-RSA is a tool that can be used in migrations studies of TKAs.

### Strengths and limitations

A major strength of this study is the randomized controlled design embedded in an ongoing clinical trial, ensuring high methodological quality and generalizability. To our knowledge, this is the first randomized controlled trial assessing the precision of CT-RSA on tibial components. Previous studies are either cadaver studies, or single-group studies with the same dosage level throughout the study [18–20]. All analyses were performed by a CTMA-certified observer (FDØ). Although the observer was not blinded, the patients, study nurse, statistician and first author (FB) were blinded for both implant type and dosage level, thus minimizing bias. Furthermore, the use of two implant designs enabled the assessment of dose effects in different geometries. In addition, our study was performed in accordance with existing recommendations for migration analysis [21–23].

Limitations include the single-centre setting and a relatively small sample size (*n* = 50), which may have limited the detection of very small differences in precision. However, this is still by far the largest study on CT-RSA on TKA to date [18, 20]. 50 patients is usually considered sufficient for a randomized controlled trial [24]. In addition, it was a precision study with double examinations under the assumption of zero motion at two follow-up points. The accuracy of CT-RSA was not measured. The fact that the double acquisitions for the low-dose and standard-dose CT-RSA protocols were performed at two different time points may introduce potential confounding. Nevertheless, each double examination was conducted within only a few minutes, and the assumption of negligible true implant motion between acquisitions is therefore considered valid, despite the two protocols being performed 12 months apart. This design was chosen to simplify the CT workflow for radiographers and thereby ensure feasibility in routine clinical practice. The published Guideline for RSA and CT-RSA implant migration measurements recommend double acquisitions in clinical studies performed at the 1-year control or earlier to prevent potential dropouts [23]. Thus, this approach is not assumed to compromise the narrow confidence intervals shown in the results, and the results should be interpreted as reliable. The original protocol was changed after the study commenced, because the CTMA software originally did not provide MTPM. In November 2022 when the initial registration in clinicaltrials.gov was performed, we could only assess the maximum translation of the 5 *predefined* peripheral points (maximum total translation, MTT), and *not* the MTPM [12, 25]. In a later update of the software, it was possible to calculate the actual MTPM. Finally, while the radiation dose was reduced to 0.01 mSv, which is the lowest value that can be recorded by our CT scanner, further optimization could be explored, including iterative reconstruction techniques and alternative scanning parameters.

### Clinical implications

Our findings indicate that low-dose CT-RSA can be adopted in clinical migration studies without loss of precision, and thereby reducing patient radiation exposure. Given its practical advantages and comparable or superior precision to RSA, CT-RSA with optimized low-dose protocols may become the preferred method for implant migration assessment in future clinical trials.

## Conclusion

The findings confirm that reducing the effective radiation dose from 0.05 mSv to 0.01 mSv does not compromise the precision of CT-RSA for tibial components in TKA. The results further support the use of low-dose CT-RSA protocols in clinical studies, enabling substantial reduction in patient radiation exposure without loss of precision.

## Data Availability

The datasets generated and/or analysed during the current study are not publicly available but are available from the corresponding author on reasonable request.
